# DWI Reversibility in Acute Ischemic Stroke Due to Basilar Artery Occlusion Following Successful Recanalization

**DOI:** 10.1007/s00062-025-01512-9

**Published:** 2025-03-31

**Authors:** Niclas Launhardt, Jessica Jesser, Dimah Hasan, Rebecca May, Omid Nikoubashman, Martin Wiesmann, Thanh N. Nguyen, Markus A. Möhlenbruch, Julius Kernbach, Charlotte S. Weyland

**Affiliations:** 1 Department of Neuroradiology, University Hospital Aachen, Aachen, Germany; 2 Department of Neuroradiology, University Hospital Heidelberg, Heidelberg, Germany; 3 Department of Neurology, Boston Medical Center, Boston, USA

**Keywords:** Diffusion Magnetic Resonance Imaging, Ischemic Stroke, Basilar Artery, Magnetic Resonance Imaging, Brainstem

## Abstract

**Purpose:**

Diffusion Weighted Imaging (DWI) represents the infarct core in acute ischemic stroke. DWI reversibility is a phenomenon reported for the anterior circulation affecting small brain areas of the white matter. This study aims to define DWI reversibility in the posterior circulation after successful recanalization of basilar artery occlusion (BAO) and its influence on patient outcome.

**Methods:**

This was a retrospective analysis of two tertiary stroke-centers analyzing stroke patients between January 2015 and December 2022. Inclusion criteria were available MRI before and after acute stroke treatment and successful BAO recanalization. Brain areas were defined as brainstem, cerebellum and supratentorial brain areas supplied by the posterior circulation. These areas were compared in univariate analysis. Secondarily, patient outcome was compared between patients with DWI reversibility and patients without in univariate analysis with good outcome as primary endpoint (mRS 90d 0 to 2).

**Results:**

In total, 5/28 of included patients (21.74%) showed DWI reversibility, which was exclusively found in the brainstem. The overall extent of brainstem infarction correlated better with patient outcome compared to cerebellar or supratentorial infarction (Spearman’s ρ = 0.757; *p* < 0.001). Good outcome was more frequent in patients with DWI reversibility compared to those without (mRS 0–2, DWI+ *n* = 4, 80% vs. DWI− *n* = 6, 26%, *p* = 0.023).

**Conclusion:**

DWI restriction reversibility was observed in the brainstem of acute stroke patients with BAO. In this study, patient outcome correlates stronger with the extent of brainstem infarction compared to cerebellar or supratentorial infarction.

## Introduction

Endovascular stroke treatment (EVT) has become a standard treatment for posterior circulation acute ischemic stroke (AIS) caused by basilar artery thrombosis. Magnetic resonance imaging (MRI) is a well-established technique to evaluate the eligibility of AIS patients for EVT. Key MRI sequences for identifying eligible patients include diffusion-weighted imaging (DWI), which reflects the infarct core in anterior and posterior circulation stroke [1–3]. In patients with anterior circulation large vessel occlusions, small diffusion restrictions in the white matter and cortical areas have been shown to be reversible after successful acute therapy [2]. The reversibility of such diffusion weighted imaging restrictions in the posterior circulation, especially in infratentorial areas, has not been investigated. Case reports hint towards the possibility of reversible diffusion restrictions in the posterior circulation [4–10]. This study aims to assess the phenomenon of diffusion restriction reversibility in patients with acute ischemic stroke due to basilar artery occlusion (BAO) following successful acute stroke therapy. Primarily, the phenomenon of DWI reversibility is studied according to the brain area supplied by the basilar artery (cerebellum, brain stem and supratentorial areas). Secondly, the influence of DWI reversibility on patient outcome after acute stroke due to BAO and successful treatment is analyzed.

## Methods

### Study Design and Patient Population

This was a retrospective analysis of patients with AIS due to BAO and available MR imaging before and after acute treatment including intravenous thrombolysis and EVT. Two study sites contributed to the analysis, University Hospital Aachen and University Hospital Heidelberg. All patient presenting with an acute ischemic stroke due to basilar artery occlusion and available MRI before and after acute stroke treatment were included in the analysis in the treatment period of January 2015 to December 2022. Patients without successful recanalization of the basilar artery after i.v. thrombolysis and EVT or one of both treatment forms alone were excluded. The first study endpoint was the volume of DWI-reversibility in the different brain areas of basilar artery supply—cerebellum, brainstem and supratentorial areas. The second endpoint was the patient outcome after acute ischemic stroke due to BAO and acute treatment as per the modified Rankin Scale (mRS) at discharge and 90 days after stroke onset.

### Lesion Segmentation

The pre- and post-MRI examinations including FLAIR- and DWI-sequences were uploaded as DICOM files from the local image storage systems of the University Hospitals in Aachen and Heidelberg, pseudonymized, then stored on a local data storage system. These files were converted into NIFTI format using the “MRIcron” program (version 1.0.20201102, September 2019) [12]. The internal mode—import “DICOM to NIFTI”—was used to convert the DICOM files into NIFTI files. The images were displayed on an examination PC (Apple Macbook Air 13 inch, 2019, MacOS 14.2.1) using an image viewer.

Ischemic lesions in FLAIR and DWI were measured and segmented manually using the “ITK-Snap” program (version 4.0.2, September 2023) as several studies have confirmed the superiority of manual segmentation over automated methods [13]. We differentiated the segmentations into three distinct areas: the brainstem, cerebellum and supratentorial brain parenchyma of the posterior circulation (thalami and occipitotemporal lobes). In the pre- and post-treatment MRI, the visually identified DWI signal hyperintensities, which also showed ADC and/or FLAIR correlates, were segmented. Additionally, individual FLAIR signal hyperintensities in the post-treatment MRI were segmented. The selection of weightings corresponded to the standard MRI stroke diagnostic protocols of the participating university hospitals. The Slice thickness was between 3 to 5 mm in all patients. ADC and FLAIR weightings were also manually segmented to control and filter for T2 shine-through effects. If FLAIR images were missing, T2 weighted images were used as substitutes.

The segmented lesions were uniformly scaled to the voxel size of the FLAIR weighting and exported as NIFTI files. The segmented datasets were confirmed by two board-certified, experienced neuroradiologists with over nine years of MRI reading experience to exclude false segmentations. The two neuroradiologists examined DWI restriction to verify differentiating ADC values between affected tissue and unaffected brain tissue.

### Volumetry

For volumetry, we combined the post-interventional lesion representations from the FLAIR and DWI images using the 3D-Slicer program (version 5.6.2) to compare the volumes of the ROIs and identify volume differences [14]. The DWI imaging volume was adjusted to the FLAIR imaging volume to proceed. To visualize the reversible diffusion restriction per brain areas, we subtracted the post-interventional brain regions from the pre-interventional brain regions, allowing us to determine the location and volume of the reversible areas.

### ADC-value Calculation

ADC values were calculated for all patients of all brain regions with diffusion restriction using ITK-Snap. These ADC values were compared between the reversible and non-reversible groups, as well as with available ADC values in the anterior circulation by literature research.

### Statistical Analysis

Data were collected in Microsoft Excel (version 16.82) and analyzed using JASP (version 0.18.3 Intel) [15]. Descriptive statistics summarized clinical and radiological variables. Normal distribution was tested using the Shapiro Wilk test. Mann-Whitney U tests were performed to analyze ordinal variables like time intervals from stroke onset to recanalization, patient age, admission NIHSS, pre-stroke and follow-up mRS. Categorical variables were analyzed with Chi-square tests.

We calculated the possible correlation between the affected lesion area (brainstem, cerebellum, or supratentorial) and the outcome of the patients (measured by the mRS at 90 days) using Spearman’s correlation. For each lesion area, we determined both Spearman’s rho and the *p*-value and compared them with each other.

## Results

In total, 28 patients from two stroke centers were included in the analysis. Among them, 5 patients (21.74%) exhibited DWI reversibility on post-interventional MRI. Patients with and without DWI reversible lesions showed no difference in time metrics as time from stroke, onset to hospital admission and no differences in baseline characteristics such as age or stroke comorbidities—see Table 1.

**Table 1:** Demographic, interventional and imaging parameters of patients with acute ischemic stroke and basilar artery occlusion eligible for acute stroke treatment—comparison of patients with DWI reversibility and without DWI reversibility

	DWI reversibility+ (*n* = 5)	DWI reversibility− (*n* = 23)	*p*-value
Age, median (IQR)	63 (20.5)	69 (14)	0.471
Sex, male; *n* (%)	4 (80%)	15 (65.22%)	0.521
Wake-up stroke; *n* (%)	1 (20%)	10 (43.48%)	0.330
Posterior-circulation ASPECTS on imaging before EVT, median (IQR)	7 (1)	7 (1)	1
Time in minutes from symptom onset to recanalization, median (IQR)	701 (26)	701(591)	0.673
Time in minutes from symptom onset to groin puncture, median (IQR)	318 (49)	557 (619)	0.239
Only intravenous. thrombolysis; *n* (%)	1 (20%)	3 (13%)	0.687
NIHSS admission, median (IQR)	4 (10)	15 (19)	0.197
Pre-stroke mRS, median (IQR)	0 (0) (1 missing)	0 (1) (5 missing)	1
*Comorbidities; n (%)*			
Diabetes mellitus Type 2	0 (0%)	3 (12%)	0.393
Arterial hypertension	4 (80%)	22 (88%)	0.687
Coronary artery disease	0 (0%)	4 (16%)	0.314
Dyslipidemia	1 (20%)	6 (24%)	0.776
Atrial fibrillation	0 (0%)	4 (16%)	0.314
*Interventional details of EVT*			
Number of thrombectomy attempts; median (IQR)	2 (0.5)	2 (2)	0.936
Intracranial stenting during EVT	1 (20%)	6 (24%)	0.776
Stent-retriever thrombectomy as first approach; *n* (%)	4 (80%)	11 (47.83%)	0.191
Contact aspiration as first approach; *n* (%)	0 (0%)	6 (24%)	0.154
Combination as first approach; *n* (%)	0 (0%)	3 (12%)	0.494
*Stroke etiology; n (%)*			
Atherosclerosis	1 (20%)	8 (34.78%)	0.521
Cardio-embolic	1 (20%)	4 (17.39%)	0.890
Other causes	0 (0%)	2(8.7%)	0.494
Embolic stroke of unknown source (ESUS)	0 (0%)	7 (30.43%)	0.198
Unknown (work-up not completed)	2(60%)	2 (8.7%)	*0.020*
*Clinical outcome 90 days after stroke onset per modified Rankin Scale (mRS), n (%), Missing values: 0*			
mRS 0‑2	4 (80%)	6 (26.09%)	*0.023*
mRS 3‑4	0 (0%)	5 (21.74%)	0.25
mRS 5‑6	1 (20%)	12 (52.17%)	0.191

**Fig. 1:**
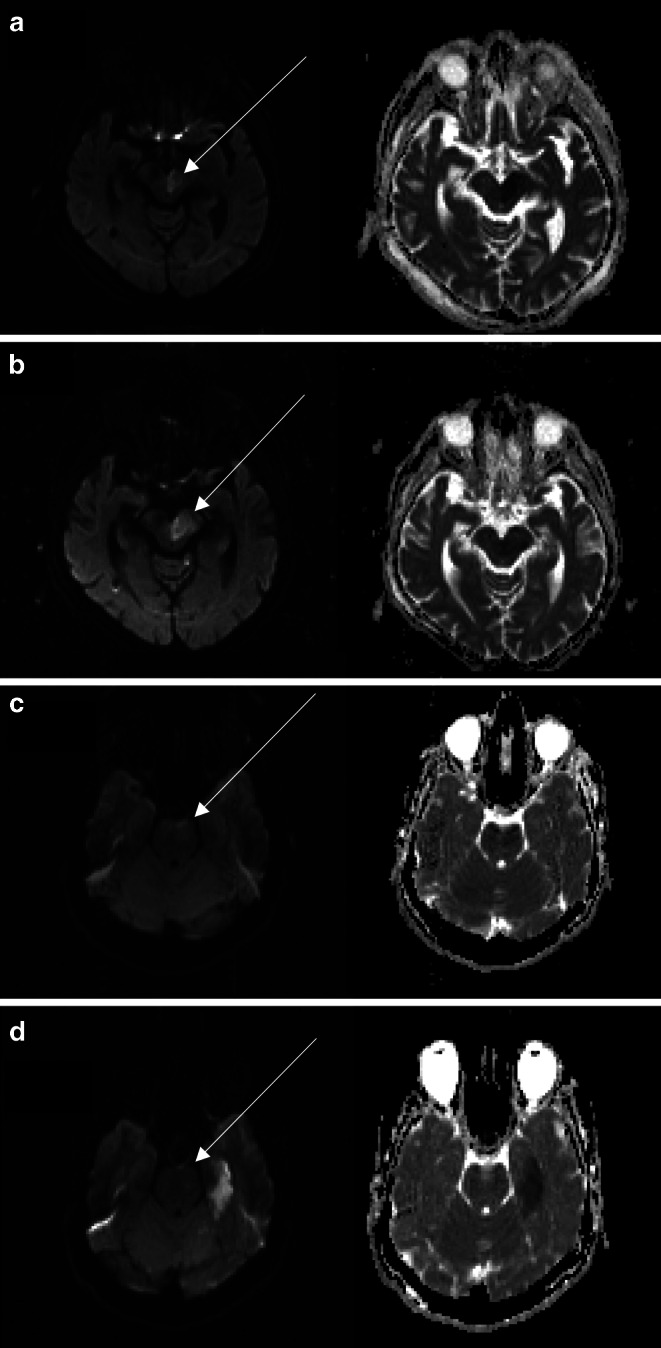
patient with acute ischemic stroke (AIS) due to basilar artery occlusion (BAO) DWI imaging b1000 image and correlating ADC Map before (**a**) and after (**b**) successful recanalization of the basilar artery: this patient showed no DWI lesion reversibility, but instead a growing left-sided mesencephalic infarction (arrows) The second patient with AIS due to BAO before (**c**) and after successful recanalization (**d**)—this patient showed a reversible left-sided pontin DWI lesion after recanalization and new or growing ischemic cerebellar and left mesotemporal lesions (arrows)

**Fig. 2:**
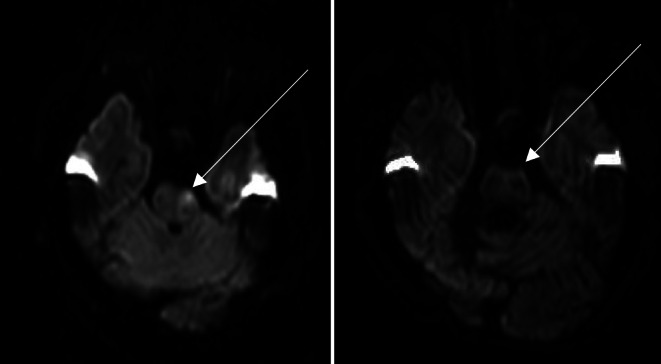
patient with acute ischemic stroke (AIS) due to basilar artery occlusion (BAO) DWI imaging b1000 image before (**a**) and after (**b**) successful recanalization of the basilar artery: this patient showed a big left pontine diffusion restriction and mRS at admission of 5. After recanalization, the diffusion restriction was completely reversible. The mRS improved from 5 to 2 after 90 days

**Fig. 3:**
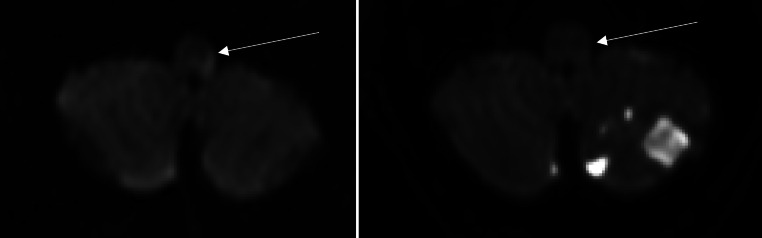
patient with acute ischemic stroke (AIS) due to basilar artery occlusion (BAO) DWI imaging b1000 image before (**a**) and after (**b**) successful recanalization of the basilar artery: this patient showed a left diffusion restriction in the medulla oblongata and a mRS at admission of 3. After recanalization, the diffusion restriction was completely reversible. The mRS after 90 days improved from 3 to 1

Reversibility of the diffusion lesion was consistently observed in the brainstem, particularly in the pons and medulla oblongata (Figs. 1, 2 and 3). There was no diffusion restriction reversibility detected in the cerebellum or supra-tentorial areas supplied by the posterior circulation. The initial DWI volume was comparable between patients with DWI reversibility (DWI+) and patients without DWI-reversibility (DWI−)—see Table 2.

**Table 2:** Comparison of ischemic lesion volumes distributed by posterior circulation (“area”)—cerebellum, brainstem and supratentorial posterior circulation “areas”

	Cerebellum	Supratentorial	Brainstem study cohort	Brainstem DWI+ vs. Brainstem DWI−
Initial DWI ischemic lesion volume, mean (IQR1–3) [mm^3^]	10883.11 (0.0–2952.75)	2864.86 (0–629.95)	1370.37 (154-95-210.0)	1241.12 (447.6–2014) vs. 1293.47 (0.0–2202.5), *p* = 0.739
Final DWI ischemic lesion volume, [mm^3^] mean (IQR1–3)	30242.56 (2934.18–30890.15)	11484.98 (0.0–5238.66)	6552.19 (550.38–9883.53)	542.16 (135.85–608.22) vs. 7858.72 (1625.21–11126.7), *p* *=* *0.014*
Incidence of DWI reversibility (yes/no), *n* (%)	0	0	5 (21.7)	–
Volume of DWI reversibility lesion, [mm^3^] mean (IQR1/3), %	0	0	698.96 (145.71–476.78), 26.4	

On average, the area of diffusion restriction in the brainstem decreased by 349.2 mm^3^ (26.4%) in patients exhibiting DWI-reversibility, resulting in a final infarct volume of 542.16 mm^3^ (IQR1/3: 135.85/608.22 mm^3^), a statistically significant difference compared to patients without DWI-reversibility (*p* = 0.008). In contrast, the final infarct volume in the non-reversible group increased from 1398.47 to 6561.65 mm^3^ (Table 2). In the cerebellum or supratentorial regions no DWI-reversibility was detected. The diffusion-restricted brain areas increased by an average of 19,359.45 mm^3^ in the cerebellum and 8620.12 mm^3^ (2864.86 mm^3^ before treatment) in the supratentorial brain areas.

Patients showing DWI reversibility (DWI+ group) were more likely to experience a good clinical outcome compared to patients without DWI reversibility (mRS 90d 0‑2, DWI+ *n* = 4/5, 80% vs. DWI− *n* = 6/23, 26%, *p* = 0.023) (Table 2). If patients exhibited DWI reversibility, their likelihood of having a good outcome (mRS 0‑2) was significantly higher compared to when no DWI reversibility was present (OR 2.428, *p* = 0.024). The difference between mRS at discharge and 90 days after discharge also suggests an improved clinical outcome in the DWI+-Group (∆mRS, median (IQR), DWI+ = −2(2) vs. DWI− = 0(0), *p* = 0.152). Only one patient with DWI-reversibility had a follow-up mRS of 3 or higher or a follow-up NIHSS at discharge of 9 or higher.

When analyzing the correlation of infarct volume and patient outcome as per mRS 90 days after stroke onset, the brainstem infarct volume correlated with patient outcome. Compared to other brain areas (cerebellum and supratentorial areas), the brainstem infarct volume correlated better with the patient outcome (brainstem ρ = 0.757; *p* < 0.001; cerebellum: ρ = 0.536; *p* = 0.003 and supratentorial: ρ = 0.395; *p* = 0.038)—see Fig. 2.

The mean ADC values varied across the affected brain regions. Reversible brainstem regions exhibited a mean ADC value of 628.43 × 10⁻^6^ mm^2^/s (IQR_1_/_3_: 583.88/597.72), whereas non-reversible brainstem regions had a mean ADC value of 490.91 × 10⁻^6^ mm^2^/s (IQR_1_/_3_: 466.41/520.5). The cerebellar regions with diffusion restriction showed a mean ADC value of 554.35 × 10⁻^6^ mm^2^/s (IQR_1_/_3_: 502.07/622.29), while the supratentorial regions with diffusion restriction had a mean ADC value of 525.77 × 10⁻^6^ mm^2^/s (IQR_1_/_3_: 469.21/571.67).

## Discussion

This study assessed DWI ischemic lesion reversibility in patients with successful treatment of acute ischemic stroke due to basilar artery occlusion. The analysis differentiated between the brain areas supplied by the basilar artery. DWI reversibility was found only in the brainstem and in no other brain area supplied by the posterior circulation (cerebellum or supratentorial areas). Notably, no DWI reversibility was observed in the cerebellar or supratentorial areas, highlighting the need for further investigation into regional differences regarding tissue characteristics within the posterior circulation. This regional differences could be due to alternating tissue resistance to ischemia, which are still not fully understood. In this study cohort, DWI reversibility in the brainstem following BAO was not uncommon, occurring in 22% of patients after successful recanalization. The observation of this study, that DWI reversibility was restricted to the brainstem, is likely due to the anatomical and physiological characteristics of the brainstem, which has a higher proportion of white matter containing fiber tracts, similar to regions in the anterior circulation where DWI restriction reversibility has been reported [2, 3, 16, 17]. In anterior circulation stroke patients, the meta-analysis of Nagaraja et al. showed that DWI restriction reversibility was found in a comparable percentage of cases (26%) [18].

Patients with DWI reversibility were more likely to experience good clinical outcome. This study showed that clinical improvement can occur in patients without DWI reversibility, attributable to the well-known fact that multiple factors contribute to patient recovery following stroke intervention. Thus, imaging biomarkers, while valuable, should be interpreted with caution and alongside clinical assessments to guide treatment and rehabilitation decisions after acute stroke treatment. Additionally, current research suggests that in the anterior circulation, the level of ADC appears to be a significant marker for the reversibility of DWI abnormalities. A cut-off value for reversibility is reported to be > 520 × 10–6 mm^2^/s [19]. Our study suggests that reversible areas in the posterior circulation exhibit similar ADC values. These regions demonstrated a mean ADC value of 628.43 × 10⁻^6^ mm^2^/s. The minimum ADC value in reversible brainstem areas was 547.12 × 10⁻^6^ mm^2^/s, close above the reported cut-off value of 520 × 10⁻^6^ mm^2^/s. In contrast, non-reversible brainstem DWI lesions presented a mean ADC value of 490.91 × 10⁻^6^ mm^2^/s. One patient exhibited an ADC value of 543.56 × 10⁻^6^ mm^2^/s but did not show reversibility. This might be due to a time delay of 338 min between MRI imaging and recanalization. No signs of DWI restriction reversibility were observed in cerebellar or supratentorial brain areas. Furthermore, ADC values in those areas did not provide significant insight into this phenomenon, as the mean ADC value was 525 × 10⁻^6^ mm^2^/s in cerebellar regions and 554.35 × 10⁻^6^ mm^2^/s in supratentorial regions. Consistent with findings in the anterior circulation, we suggest that a high rate of white matter and fiber tracts plays a crucial role in regions with possible DWI reversibility making this phenomenon more likely in the brainstem than in cerebellar or supratentorial regions. Further research with larger study cohorts is necessary to validate the ADC cut-off value for posterior circulation ischemic stroke. The brainstem infarct size after acute stroke treatment correlated better with the patient’s outcome than the infarct size of the other posterior circulation brain areas. As the results and subsequent statistical analysis demonstrate, patient outcomes can be explained by brainstem lesions in over 75% of cases, a finding that is statistically significant (rho = 0.757, *p* < 0.001). In contrast, the cerebellum appears to influence outcomes in only about 54% of cases and supratentorial lesions in only 39%. This underscores the critical importance of brainstem lesions and timely treatment, as reversibility was only observed in brainstem areas, which was closely associated with significantly improved outcomes. This finding can guide patient rehabilitation measurements after the acute treatment time and underlines the importance of brain stem lesions for the clinical outcome.

Due to study limitations such as small sample-size, retrospective design, lack of follow-up data, and mixed treatment cohorts, further research is needed to confirm these findings. As reported by Rafael-Patiño et. al., the choice of MRI protocols also might have a significant impact on the pathologies depicted in MRI scans, rendering multicenter studies challenging to reproduce [20]. Manual segmentation is considered the most effective method for visualizing lesions in MRI imaging. However, DWI-hyperintensity potentially does not fully depict true reversibility, as DWI imaging inherently exhibits slight variations between individual image acquisitions. Two-fold imaging reading by two neuroradiologists was performed to determine notable DWI-hyperintensity as in DWI-restriction and DWI-restriction reversibility, but the ground truth of “true” diffusion restriction might not be completely in line with DWI imaging, although the ADC value is a quantifiable imaging parameter. Clinicians rely on DWI to define ischemic lesions. Thus, this study might contribute in raising awareness of DWI reversibility in the brainstem when it comes to defining the extent of ischemic damage a patient might expect. This is especially important, when taking into account, that brainstem infarcts might define the outcome more than cerebellar or supratentorial infarcts as this study suggests. While this study was able to show that DWI reversibility might be restricted to the brainstem in posterior circulation stroke, future studies should focus on larger patient cohorts, standardized MRI protocols and long-term clinical outcomes to provide a more comprehensive understanding of DWI reversibility in patients with basilar artery occlusion.

## Conclusion

This study shows that DWI restriction reversibility in patients with acute ischemic stroke due to basilar artery occlusion might be a common phenomenon exclusively found in the brainstem. Patients with DWI reversibility had a higher rate of good clinical outcome compared to patients without. In this study, patient outcome correlated stronger with the extent of brainstem infarction compared to cerebellar or supratentorial infarction.

## Abbreviations

ADC: Apparent diffusion coefficient
BAO: Basilar artery occlusion
DWI: diffusion weighted imaging
EVT: Endovascular therapy
FLAIR: Fluid-attenuated inversion recovery
mRS: Modified Ranking Scale
NIHSS: National Institution of Health Score scale
